# The mTOR inhibitor Rapamycin protects from premature cellular senescence early after experimental kidney transplantation

**DOI:** 10.1371/journal.pone.0266319

**Published:** 2022-04-21

**Authors:** Uwe Hoff, Denise Markmann, Daniela Thurn-Valassina, Melina Nieminen-Kelhä, Zulrahman Erlangga, Jessica Schmitz, Jan Hinrich Bräsen, Klemens Budde, Anette Melk, Björn Hegner

**Affiliations:** 1 Department of Nephrology and Critical Care Medicine, Charité – Universitätsmedizin Berlin, Freie Universität Berlin, Humboldt-Universität zu Berlin, Berlin Institute of Health, Berlin, Germany; 2 Nieren- und Dialysezentrum Schöneberg-Tempelhof, Berlin, Germany; 3 Childrens’ Hospital, Hannover Medical School, Hannover, Germany; 4 Departement of Neurosurgery, Charité – Universitätsmedizin Berlin, Freie Universität Berlin, Humboldt-Universität zu Berlin, Berlin Institute of Health, Berlin, Germany; 5 Nephropathology Unit, Institute of Pathology, Hannover Medical School, Hannover, Germany; 6 Vitanas Hospital for Geriatric Medicine, Berlin, Germany; Universidade de Sao Paulo, BRAZIL

## Abstract

Interstitial fibrosis and tubular atrophy, a major cause of kidney allograft dysfunction, has been linked to premature cellular senescence. The mTOR inhibitor Rapamycin protects from senescence in experimental models, but its antiproliferative properties have raised concern early after transplantation particularly at higher doses. Its effect on senescence has not been studied in kidney transplantation, yet. Rapamycin was applied to a rat kidney transplantation model (3 mg/kg bodyweight loading dose, 1.5 mg/kg bodyweight daily dose) for 7 days. Low Rapamycin trough levels (2.1–6.8 ng/mL) prevented the accumulation of p16^INK4a^ positive cells in tubules, interstitium, and glomerula. Expression of the cytokines MCP-1, IL-1β, and TNF-α, defining the proinflammatory senescence-associated secretory phenotype, was abrogated. Infiltration with monocytes/macrophages and CD8^+^ T-lymphocytes was reduced and tubular function was preserved by Rapamycin. Inhibition of mTOR was not associated with impaired structural recovery, higher glucose levels, or weight loss. mTOR inhibition with low-dose Rapamycin in the immediate posttransplant period protected from premature cellular senescence without negative effects on structural and functional recovery from preservation/reperfusion damage, glucose homeostasis, and growth in a rat kidney transplantation model. Reduced senescence might maintain the renal regenerative capacity rendering resilience to future injuries resulting in protection from interstitial fibrosis and tubular atrophy.

## Introduction

Despite improvements in short-term outcomes after kidney transplantation due to lower rates of acute rejection little progress has been made with regard to allograft half-life beyond the first year after transplantation [1]. Interstitial fibrosis and tubular atrophy (IF/TA) as well as glomerulosclerosis, the key histological features of ‘chronic allograft nephropathy’, represent a final common pathway of multiple immunological and non-immunological injury mechanisms with the consequence of deteriorating graft function culminating in transplant failure [2]. Several layers of evidence have linked IF/TA and allograft failure to accumulation of renal cells that had acquired a specific cell fate called premature cellular senescence [3,4].

Senescent cells are characterized by an irreversible cell cycle arrest that correlates with high expression levels of cell cycle inhibitors such as p16^INK4a^, p21^CIP1^, and p27 [5]. Moreover, they remain metabolically active, are resistant to apoptosis, and can secrete a plethora of proinflammatory cytokines, chemokines, and growth factors constituting the senescence-associated secretory phenotype (SASP) acting in an autocrine and paracrine manner [3,5]. The SASP implicates senescent cells not only as supporters of inflammation by secretion of tumor necrosis factor-α (TNF-α), monocyte chemoattractant protein-1 (MCP-1), and others but also as important mediators of tissue remodeling via matrix metalloproteinases, platelet-derived growth factor AA, and transforming growth factor-β [3–5]. Increased expression of senescence markers, particularly of p16^INK4a^, is correlated with age and IF/TA due to both native chronic kidney disease or allograft damage [3,6,7]. Furthermore, the amount of p16^INK4a^ expression in implantation biopsies can predict the outcome of kidney transplantation [8,9]. Many well-established risk factors for the development of IF/TA in transplanted kidneys and graft failure have also been linked to the induction of premature cellular senescence. Non-immunological determinants include higher donor age [10], hypertension [11], diabetes mellitus [12], and ischemia/reperfusion injury (IRI) [4]. But also calcineurin inhibitor (CNI) toxicity [13,14] and immunological insults such as acute and chronic cellular and antibody mediated rejection can be involved [15–17]. Important mechanisms operative in the aforementioned situations that accelerate the transition to a senescent phenotype apart from physiologic aging are DNA damage, inflammation, the unfolded protein response, and oxidative stress [5,17].

On the contrary, reduced signaling via the mechanistic target of Rapamycin (mTOR) by means of caloric restriction or pharmacological agents such as the macrolide Rapamycin (Rapa) has been shown to interfere with the development of cellular senescence [18–21] and can even increase life span consistently in all model organisms studied so far [22,23]. Moreover, Rapa can counteract SASP expression via downregulation of interleukin-1α (IL-1α) [24] and SASP mediated effects since many of its constituents rely on mTOR signaling [5].

Rapa also features potent immunosuppressive properties derived from its influence on a large set of immune cells [25] and has been approved as an immunosuppressant after organ transplantation in 1999. Since then, Rapa and the subsequently developed mTOR inhibitor (mTORi) everolimus have been associated with the hope to effectively reduce IF/TA and improve long-term function of renal allografts by reduction, elimination, or avoidance of CNI and their inherent toxicity and additional unique intrinsic properties [26]. Extended cellular damage may ultimately lead to nephron loss and the histologic picture of IF/TA. It is important to note that the basis for development of IF/TA appears to be established early in the course after transplantation or even before implantation since donor age, the extent of graft damage related to brain death, preservation, and reperfusion is an important determinant of IF/TA [27]. Thus, timely strategies targeting the initiation of deleterious processes early on are most promising to substantially reduce nephron loss and improve long-term graft survival.

Here, we tested the hypothesis that mTOR inhibition with Rapa immediately after experimental allogeneic kidney transplantation in a life-supporting rat model with a low-responder strain combination can reduce cellular senescence in the graft. Rapa very potently protected all kidney compartments from accumulation of senescent cells in the peritransplant phase, inhibited the SASP-type inflammatory response, and improved functional recovery without a negative impact on glucose homeostasis and growth. Thus, early mTOR inhibition in renal transplantation holds the potential to prevent excessive conversion of kidney cells to a senescent phenotype during the vulnerable preservation/reperfusion period. This might fundamentally improve graft resilience against later injuries by safeguarding regenerative capacity leading to protection from IF/TA and functional deterioration.

## Materials and methods

### Animals and surgical procedures

Local authorities (Landesamt für Gesundheit und Soziales, LaGeSo, Berlin, Germany) approved all procedures and we followed the guidelines of the American Physiological Society as well as the ARRIVE guidelines. Male inbred Fischer (F344) and Lewis (Lew, RT1) rats (Harlan-Winkelman, Sulzbach, Germany) 10-weeks of age and weighing 190–300 g were kept at a constant temperature of 24°C under regular lighting conditions (lights on 6:00, lights off 18:00) with free accessible tap water and food (C-1000, Altromin, Lage, Germany). For surgery and organ harvesting, all rats were anesthetized with isoflurane (2% for induction, 1.5% thereafter; Abbott, Wiesbaden, Germany). Kidney grafts were prepared from Fischer rats as described previously [28]. Briefly, the vessels and the ureter were separated from fibrotic tissue. Collateral branches of the vessels were dissected by electrocauterization and the distal ureter was cut next to the bladder. Prior to ablation of the artery and the vein with microaneurysm clips and iris microscissors, kidneys were perfused in situ with 5 mL cold University of Wisconsin (UW) solution via the aorta. Kidneys were placed in UW at 4°C for 2 hours after explantation. After removing both native kidneys, the donor kidney was grafted orthotopically. The vessels and the ureter were anastomosed end-to-end with 10–0 polypropylene (Prolene, Ethicon, Norderstedt, Germany) within 30 minutes. Postoperative analgesia was achieved with 0.5 mg/mL tramadol (Grünenthal, Stolberg, Germany) in drinking water for 3 days. Animals were monitored at least once daily for clinical and behavioral signs of pain and suffering. There were no postoperative complications. Survival until the planned endpoint was 100%.

In total, 40 animals were used as experimental units. They were randomly allocated to the treatment groups without any specific randomization protocol. 19 rats were assigned to the vehicle group and 21 to receive Rapa. In the vehicle group, 5 animals were sacrificed on days 1, 2, and 5, respectively, and 4 animals on day 7. In the Rapa group, 6 rats were sacrificed on days 1 and 2, respectively, 4 rats on day 5 and 5 rats on day 7. The order of treatments, duration of observation periods, and cage location was evenly distributed across the whole study. The sample size was estimated from previous studies with the same and similar models by our group [28–33]. For Rapa treatment, the commercially available 1 mg/mL Rapa solution for oral application in patients was used (Wyeth, Madison, NJ). Rats received a loading dose of 3 mg/kg bodyweight on day 0 and daily maintenance doses of 1.5 mg/kg bodyweight thereafter by gavage. Rats in the vehicle group received an equivalent amount of water by gavage every day.

For measurement of creatinine clearance, proteinuria, and fractional excretion of sodium (FE_Na_), urine was collected over 24 h using metabolic cages.

Animals were sacrificed at the indicated time points for harvesting of the kidney grafts and collection of venous blood. All clinical chemical analyses were performed with automated measurements at the university’s core laboratory facility.

All technically valid data points were included in the analyses. No animals were excluded from the study. The following analyses were performed in a blinded manner after organ harvesting.

### Histology, immunohistochemistry, and morphometric quantification

For histology and immunohistochemistry, formalin fixed paraffin sections (4 μm) were processed following previously described protocols [32].

Immunoperoxidase staining for p16^*INK4a*^ and IL-1β were performed as described previously [34]. Briefly, sections were deparaffinized and hydrated. The sections were immersed in 3% H_2_O_2_/methanol to inactivate endogenous peroxidase. Slides were blocked with 20% normal goat serum overnight. Tissue sections were then incubated for 1 h at room temperature or overnight at 4°C with the primary antibodies (p16: mouse monoclonal antibody, Clone F-12, Santa Cruz Biotechnologies, Heidelberg, Germany, dilution 1:100; IL-1β: mouse monoclonal antibody, clone E7, Santa Cruz Biotechnologies, dilution 1:300) or the corresponding isotype control and then rinsed with PBS. Following 30 min of incubation with the Envision monoclonal system (DakoCytomation) sections were washed again in PBS. Visualization was performed using the diaminobenzidine (DAB) substrate kit (DakoCytomation). The slides were counterstained with hematoxylin and mounted. Analysis was done by counting 10 randomly chosen fields of vision (FOV; 200x magnification) by a blinded observer. The percentage of positive nuclei in comparison to total nuclei number was assessed separately for tubuli, interstitium, and glomerula in each FOV and then summarized as a mean per animal and compartment.

The alkaline phosphatase/anti-alkaline phosphatase (APAAP) complex method (DakoCytomation, Hamburg, Germany) was applied for staining with antibodies directed against ED1 (CD68; Serotec, Oxford, UK, dilution 1:1000) and CD8 (Serotec, dilution 1:100). Amino ethyl carbazole (AEC; DakoCytomation) was used as the chromogen. For staining of ED2, tissue sections were pretreated with Fast Enzyme (Zytomed Systems, Berlin, Germany) and then incubated with a mouse anti-CD163 antibody (Serotec, dilution 1:50) overnight at 4°C followed by ZytoChem Fast AP One-Step Polymer anti-mouse/rabbit (Zytomed Systems) and Permanent AP-Red-Kit (Zytomed Systems). Corresponding isotype antibodies instead of specific antibodies served as controls.

Hematoxylin counterstaining was applied to all slides after immunostaining. For quantification, 10 FOV at 400x magnification were randomly chosen. The percentage of positively stained interstitial, glomerular, or tubular cells was first separately calculated for each FOV and then summarized as a mean per animal and examined compartment.

The acute kidney injury (AKI) score was obtained from hematoxylin and eosin (H&E) stained sections. The score comprised tubular dilatation, basal membrane denudation, loss of the brush border, tubular cell flattening, tubular cell vacuolization, and tubular casts. The percentage of affected tubular cross sections was first acquired for the individual score components and finally combined by calculating the mean percentage of all components separately for each animal.

### Quantitative real-time PCR

As previously described [35], after extraction from deep frozen graft tissue samples with TRIzol (Invitrogen, Carlsbad, CA, USA) total RNA was purified and spectrometrically quantified. Reverse transcription of 1 μg RNA per sample into complementary DNA (cDNA) was done with the PCR Core Kit and random hexamer primers (Applied Biosystems, Foster City, CA, USA) in a total volume of 20 μl according to the manufacturer’s instructions.

Amplification and real-time quantification was achieved with the Light Cycler PCR and detection system (Roche, Mannheim, Germany) using specific primer pairs (TIB Molbiol, Berlin, Germany) with the following sequences: Inducible NO synthetase (iNOS), forward 5’–CTG TGT CCA ACA TGC TGC TAG AAA TTG, reverse 5’–TAA AGG TCT TCT TCC TGG TGA TGC C; MCP-1, forward 5’–AGG TCT CTG TCA CGC TTC TG, reverse 5’–TGT CAT ACT GGT CAC TTC TA; pro-IL-1β, forward 5’–CAA GCA ACG ACA AAA TCC C, reverse 5’–GAA CTG TGC AGA CTC AAA CTC C; TNF-α, forward 5’–ATG GGC TCC CTC TCA TCA GT, reverse 5’–ACT CCA GCT GCT CCT CTG CT; IL-10, forward 5’–ACT GCT ATG TTG CCT GCT CTT ACT, reverse 5’–GAA TTC AAA TGC TCT TGA TTT CT. Glyceraldehyde-3-phosphate dehydrogenase (GAPDH), forward 5’–CCA TCT TCC AGG AGC GAG AT, reverse 5’–GAT GAC CTT GCC CAC AGC CT served as the internal standard. cDNA obtained from 0.1 μg RNA was applied to each reaction together with Taq polymerase, magnesium chloride (3 mM), Master Sybr Green Mix^®^ (Roche, Mannheim, Germany), and primers in a total volume of 20 μl. Specificity of the reactions was confirmed by melting curve analyses. Semi-quantitative calculation of transcripts was performed with the delta-CT method using the program Quant V3·0. Arbitrary units (AU) were calculated with the formula $\text{AU}=\frac{1}{2^{\left(CtTarget-CtReference\right)}}\times 1000$. Thus, AU represent a semi-quantitative measure of the target gene normalized to the reference gene GAPDH.

### Statistical analysis

All results are expressed as means ± SEM. Numbers of animals are given in the legend of each figure. Two-way analysis of variance (ANOVA) with Bonferroni’s multiple comparisons test was used for comparison of the treatment groups and time points as well as the macrophage phenotype on day 7. A two-sided P value of <0.05 was considered statistically significant. All tests were performed with GraphPad Prism 5.0 (GraphPad Software, La Jolla, CA, USA) for Windows.

## Results

To test our hypothesis that mTOR inhibition with Rapa might protect from cellular senescence in the context of preservation/reperfusion injury in kidney transplants we applied low doses of Rapa to our life-supporting low-responder Fischer-to-Lewis rat kidney transplantation model [36]. Treatment was initiated on day 0. Rapa trough levels were 2.1–2.3 ng/mL on days 1 and 2 and increased to 6.5–6.8 ng/mL on days 5 and 7 (Table 1) reaching the target range of modern immunosuppressive regimens [37].

**Table 1:** Rapa trough levels.

	day 1	day 2	day 5	day 7
Rapa trough level [ng/mL]	2.14±0.17	2.32±0.40	6.83±1.28	6.48±0.88

### Rapa reduces cellular senescence and dampens the SASP-type inflammatory response

**Fig 1:**
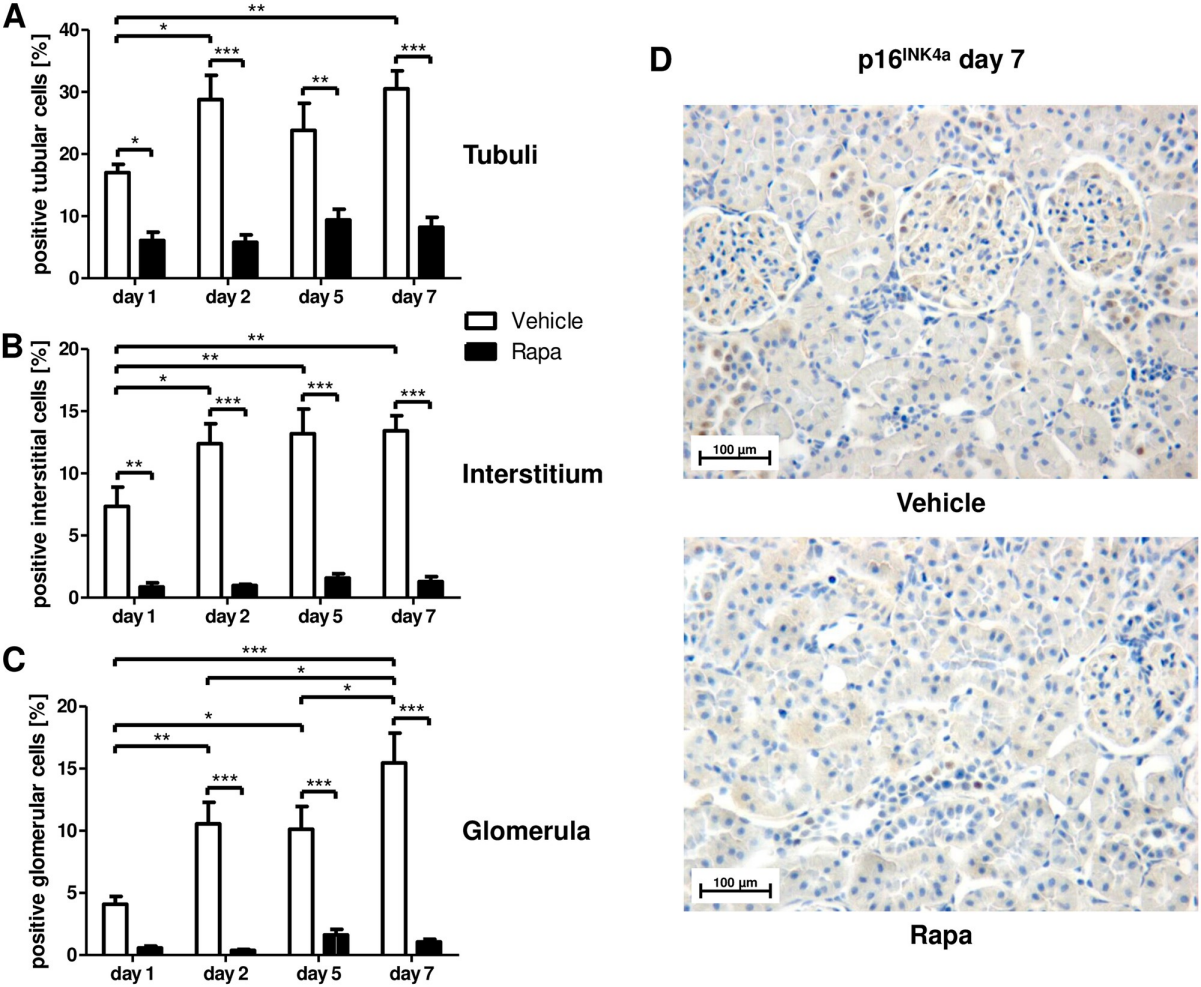
Cellular senescence in different renal compartments early after kidney transplantation in rats treated with Rapa. Immunohistochemistry for the cell cycle inhibitor p16^INK4a^ as an indicator of replicative senescence. Quantitative assessment of positive cells in tubuli (A), the interstitium (B), and glomerula (C). n = 3–6. *P<0.05, **P<0.01, ***P<0.001. (D) Representative photomicrographs from vehicle and Rapa treated animals on day 7 after transplantation.

Cellular senescence as measured by expression of p16^INK4a^ and SASP-type proinflammatory cytokines were significantly reduced by Rapa treatment. p16^INK4a^ expression was diminished in tubular and interstitial cells as early as on day 1 (Fig 1A and 1B) and in glomerula starting on day 2 (Fig 1C), while senescence increased in all compartments in vehicle treated animals on day 2 and displayed an additional increment in glomerula on day 7 low levels persisted in rats receiving Rapa (Fig 1A–1D).

**Fig 2:**
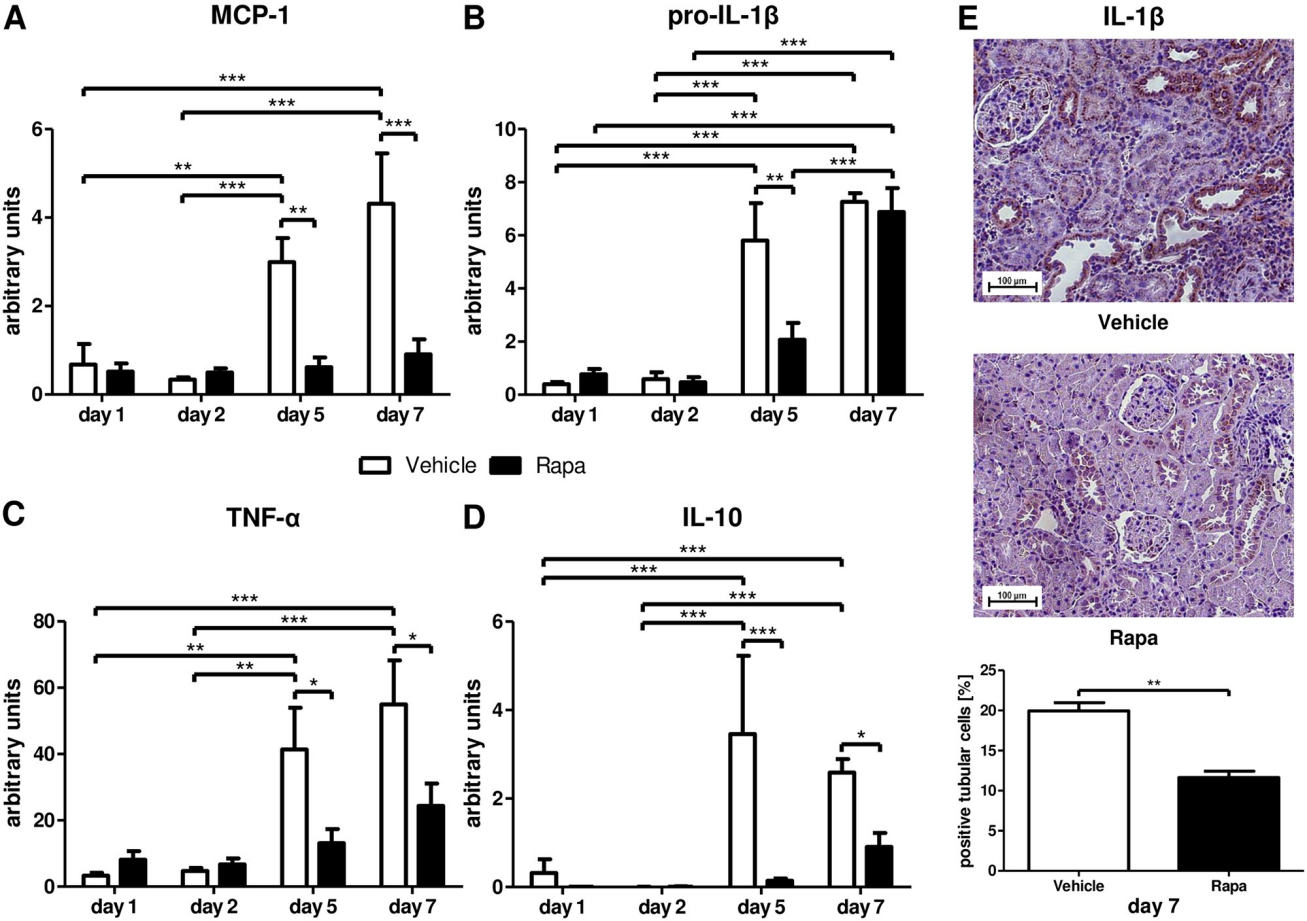
Senescence-associated secretory phenotype (SASP) related cytokines after kidney transplantation in vehicle and Rapa treated rats. Whole tissue lysates from allografts were analyzed with semi-quantitative real-time PCR for mRNA expression of the proinflammatory cytokines monocyte chemoattractant protein-1 (MCP-1; A), pro-interleukin-1β (pro-IL-1β; B), and tumor necrosis factor-α (TNF-α; C) as well as of the anti-inflammatory cytokine interleukin-10 (IL-10; D). (E) Immunohistochemistry for IL-1β on day 7. Representative photomicrographs and quantification as percentage of positive tubular cells are shown. n = 3–6. *P<0.05, **P<0.01, ***P<0.001.

Along with the reduction in p16^INK4a^, we found significant differences in signature cytokines belonging to the SASP. On days 1 and 2, only low basal levels of mRNA transcripts for the proinflammatory cytokines MCP-1, pro-IL-1β, and TNF-α as well as for the anti-inflammatory cytokine IL-10 were detected (Fig 2). MCP-1 was markedly upregulated only in the vehicle group on day 5 and thereafter (Fig 2A). Pro-IL-1β and TNF-α were also considerably upregulated in rats receiving vehicle on days 5 and 7 (Fig 2B and 2C). However, pro-IL-1β mRNA transcripts reached the same level in Rapa treated animals on day 7 (Fig 2B). IL-10, an anti-inflammatory cytokine balancing the inflammatory response, followed the same expression pattern as MCP-1 and TNF-α with increased transcription on days 5 and 7 in vehicle animals only (Fig 2D). To verify that Rapa reduces SASP cytokine production in kidney cells, we performed immunohistochemistry for IL-1β. On day 7, 20% of tubular cells in control animals expressed IL-1β in contrast to only 11.6% in Rapa treated rats (Fig 2E).

**Fig 3:**
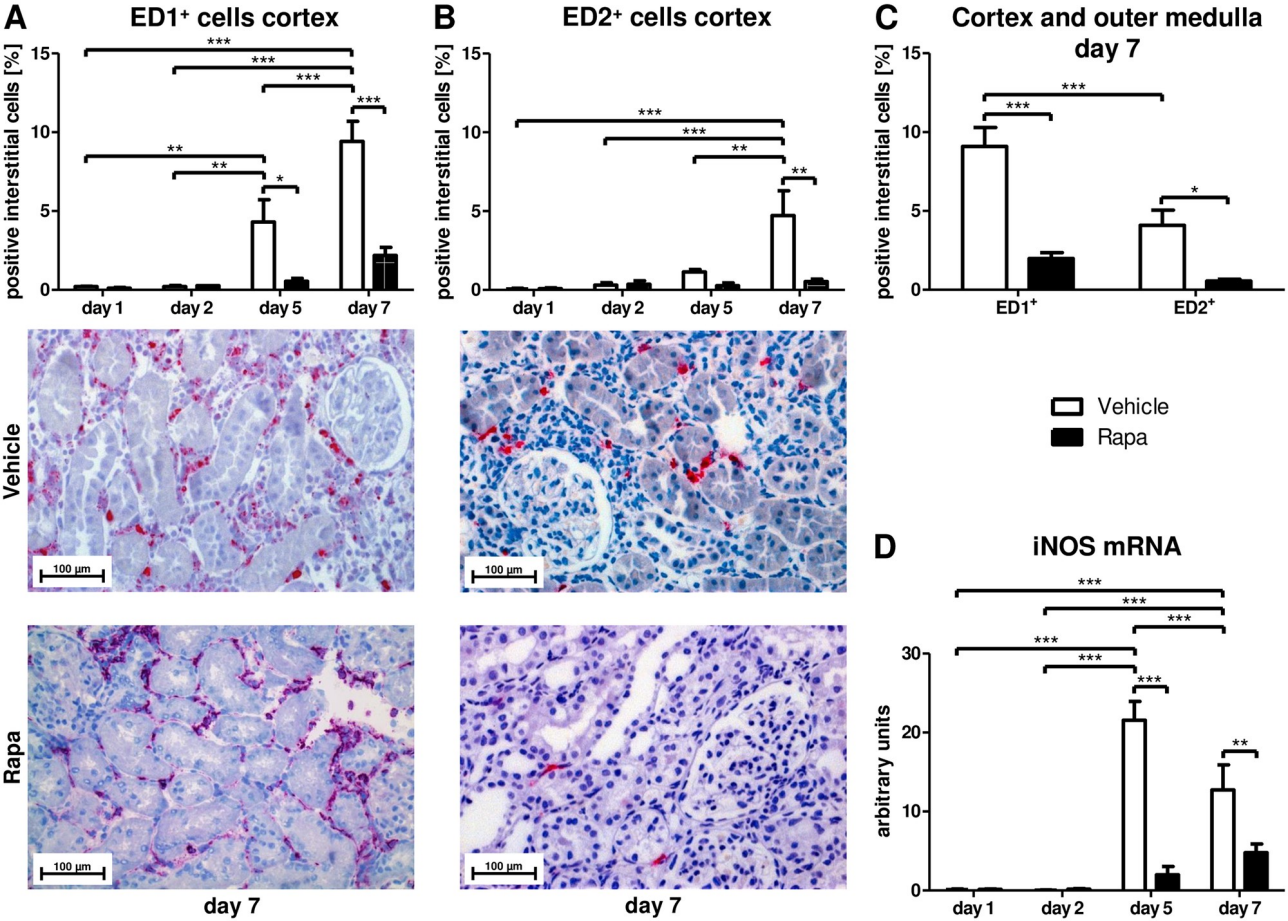
Infiltrating macrophages after kidney transplantation in vehicle and Rapa treated rats. (A) ED1 and (B) ED2 positive macrophages in the cortex calculated as percentage of positive interstitial cells by immunohistochemistry. Representative photomicrographs are shown. n = 3. (C) Comparison of ED1 and ED2 positive infiltrating cells on day 7 in vehicle and Rapa treated rats. Cortex and outer medulla were analyzed together. n = 6–8. (D) Semi-quantitative real-time PCR for mRNA transcripts of inducible NO synthase (iNOS) in whole tissue extracts from kidney allografts. n = 4–6. *P<0.05, **P<0.01, ***P<0.001.

**Fig 4:**
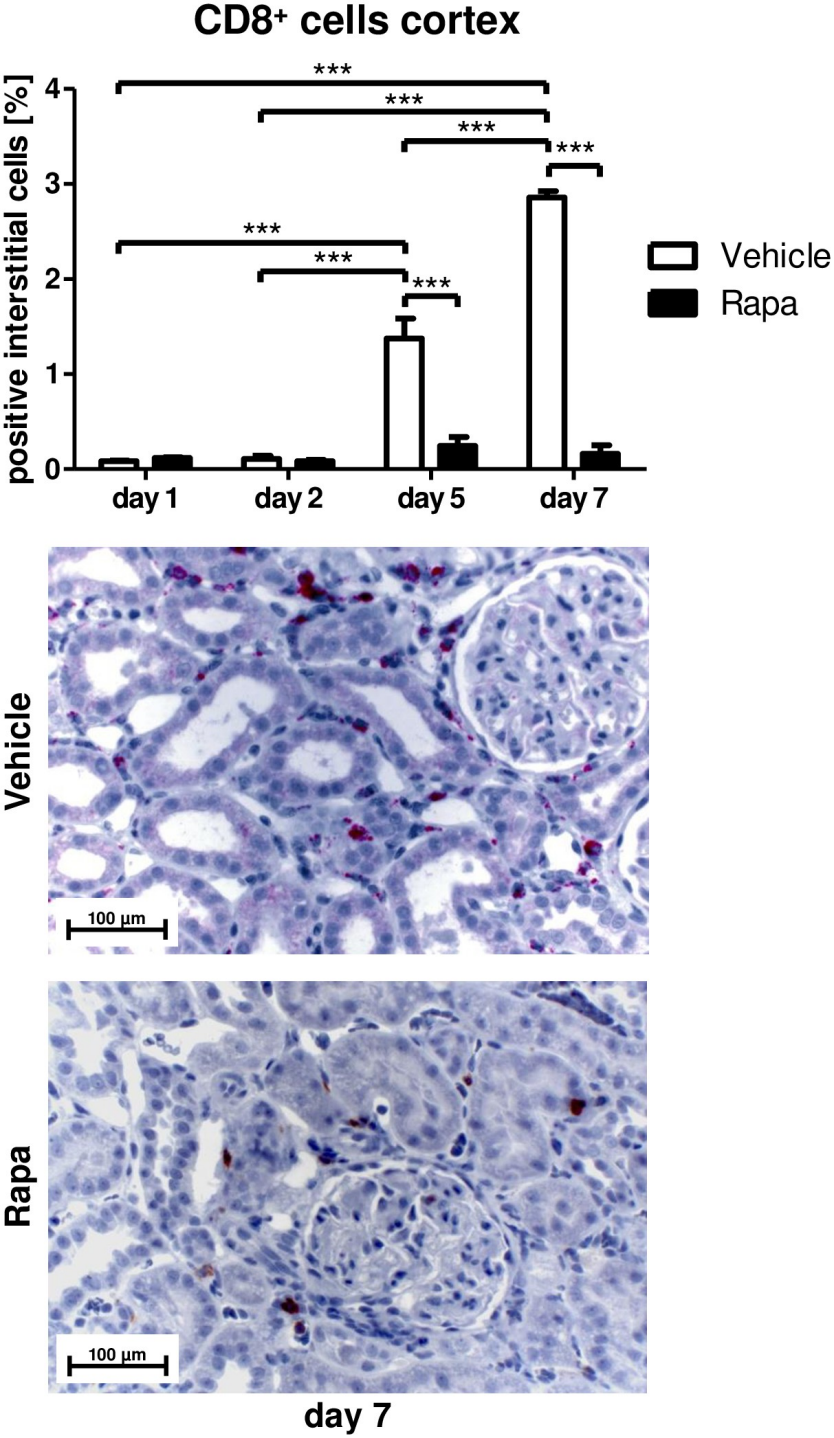
Infiltrating cytotoxic T-cells after kidney transplantation in vehicle and Rapa treated rats. CD8 positive T-cells in the cortex calculated as percentage of positive interstitial cells by immunohistochemistry. Representative photomicrographs are shown. n = 3. ***P<0.001.

While senescent cells promote inflammatory processes via the SASP, inflammation is in turn an important driver of cellular senescence by itself [5]. In addition to the SASP elements, we examined the impact of Rapa on inflammatory cell infiltration of the allografts. ED1-positive monocytes/macrophages were found in the cortex of vehicle treated rats on day 5 with a further increase on day 7 (Fig 3A) and in the outer medulla on day 7 (S1A Fig) while there was no significant increase in the Rapa group (Fig 3A and S1A Fig). ED2-positive cells representing a subset of alternatively activated macrophages with anti-inflammatory [38] and profibrotic [39] properties were detected in cortex (Fig 3B) and outer medulla (S1B Fig) on day 7 in rats receiving vehicle but not in those receiving Rapa (Fig 3B and S1B Fig). Overall, less than half of the infiltrating macrophages had M2 polarity in vehicle treated animals on day 7 (Fig 3C). To further assess polarization of macrophages, we measured iNOS mRNA expression with quantitative RT-PCR in whole tissue lysates. Only vehicle treated animals featured markedly increased mRNA levels of iNOS, a signature gene of proinflammatory macrophages, on days 5 and 7 (Fig 3C). CD8 positive T-cells infiltrated cortex and medulla of transplanted kidneys in vehicle but not in Rapa treated rats on day 5 and even more on day 7 (Fig 4 and S1C Fig) accompanying the macrophage infiltrate.

### Rapa permits recovery from preservation/reperfusion injury

**Fig 5:**
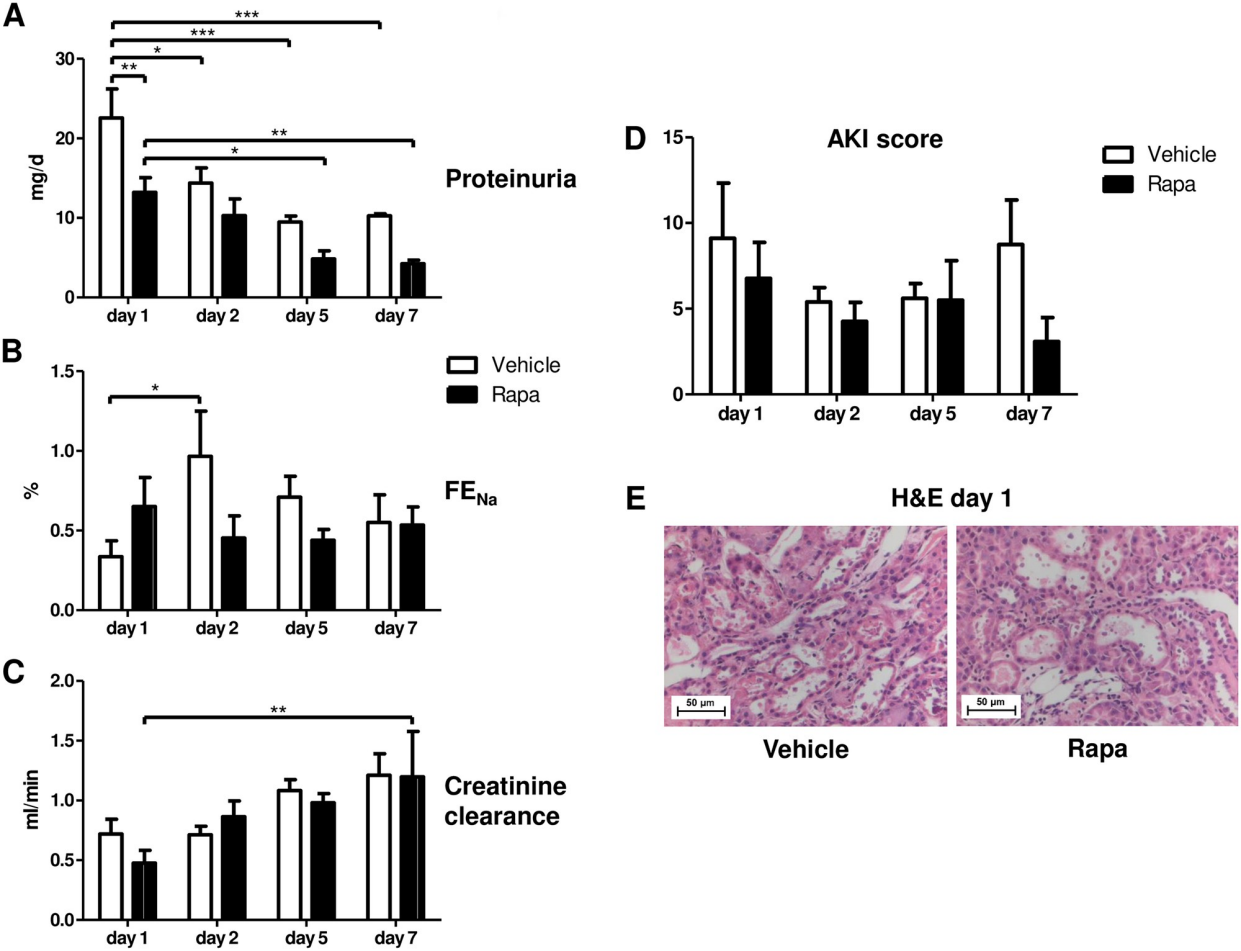
Functional and structural recovery during the first 7 days after kidney transplantation in vehicle and Rapa treated rats. (A) Proteinuria measured over 24 h. n = 4–5. (B) Fractional excretion of sodium (FE_Na_) given in %. n = 4–6. (C) Creatinine clearance calculated from a 24-h urine collection. n = 3–5. (D) Acute kidney injury (AKI) score representing the mean percentage of tubular cross sections affected with tubular dilatation, basal membrane denudation, loss of the brush border, tubular cell flattening, tubular cell vacuolization, and tubular casts. n = 3–6. *P<0.05, **P<0.01, ***P<0.001. (E) Representative photomicrographs from hematoxylin and eosin (H&E) stained kidney sections from vehicle and Rapa treated animals on day 1 after transplantation.

On a functional level, proteinuria was lower with Rapa treatment on day 1 and decreased in both groups through day 7 (Fig 5A). Fractional excretion of sodium (FE_Na_) increased only in vehicle treated animals on day 2 (Fig 5B) whereas creatinine clearance was similar in both groups (Fig 5C). These findings suggest less impaired glomerular and tubular function in animals receiving the mTORi Rapa in the early posttransplant period. However, the AKI score did not differ according to treatment (Fig 5D and 5E).

### Influence of Rapa on glucose homeostasis and growth

**Fig 6:**
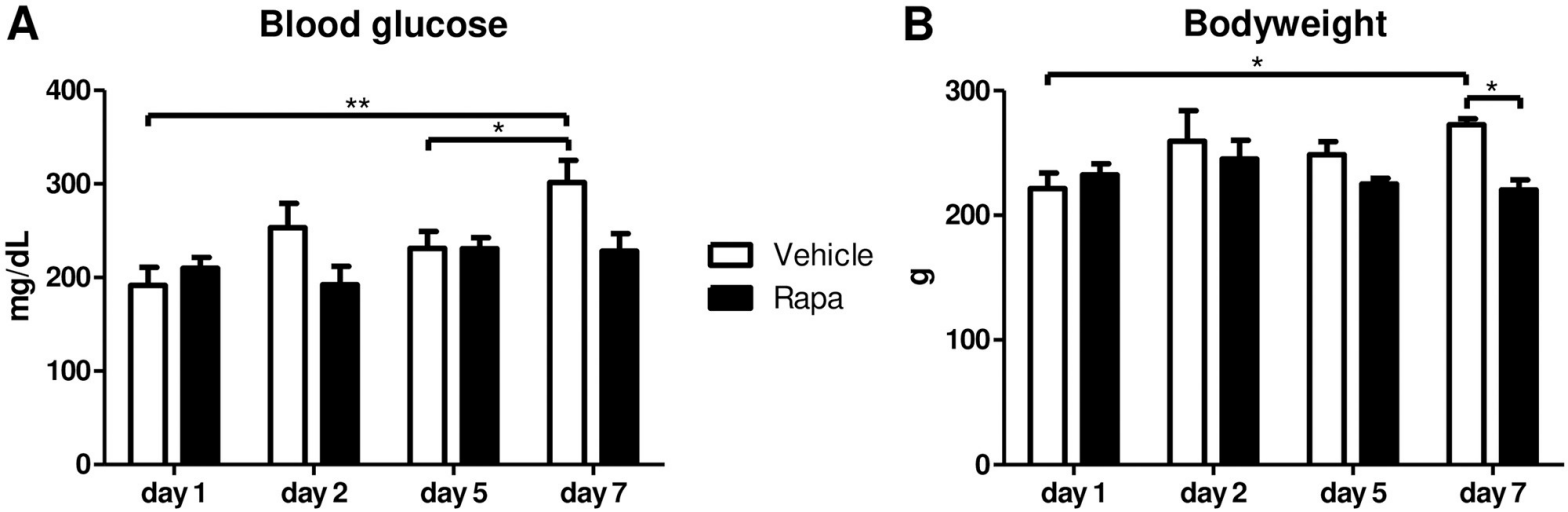
Metabolic parameters of rats treated with vehicle or Rapa after kidney transplantation. Measurements were performed at the end of the observation period on the indicated time points after transplantation. (A) Blood glucose levels. n = 3–5. (B) Body weight. n = 3–5. *P<0.05, **P<0.01.

Impaired glucose tolerance and induction of diabetes mellitus is a known adverse effect of mTORi such as Rapa [40]. As hyperglycemia has been linked to premature cellular senescence [12], we measured blood glucose levels after transplantation. Rapa treated animals did not show increased glucose concentrations throughout the observation period of one week (Fig 6A). Nevertheless, there was some increase in blood glucose levels in the vehicle group on day 7 without statistical significance in comparison to the Rapa group (Fig 6A). Furthermore, we assessed the bodyweight to monitor adverse effects of mTOR inhibition on overall growth. Bodyweight remained stable in Rapa treated rats whereas it increased by 23% until day 7 in rats allocated to vehicle (Fig 6B).

## Discussion

The most outstanding finding of our study was that mTOR inhibition with Rapa potently protected renal allografts from premature cellular senescence associated with preservation/reperfusion injury in a life-supporting kidney transplantation model in rats. Reduced senescence was accompanied by a blunted SASP-type inflammatory response, milder tubular dysfunction, and unimpeded structural and functional recovery. No adverse metabolic effects were detected.

Importantly, the immunosuppressive properties of Rapa interfere with the SASP produced by senescent cells [3,4,24,41] both upstream and downstream. In contrast to vehicle treated animals, Rapa very effectively reduced the amount of senescent cells as indicated by considerably lower numbers of p16^INK4a^ positive cells already on day 1 and throughout the whole observation period. The prevention of p16^INK4a^ expression, one of the most important senescence markers, was paralleled by markedly reduced levels of SASP-related proinflammatory cytokines resulting in almost absent inflammatory infiltrates. Of note, macrophages with profibrotic M2 polarization were equally suppressed by Rapa. On the contrary, we documented an increase in senescent cells in the vehicle group that preceded the expression of proinflammatory cytokines and graft infiltration with inflammatory cells. Moreover, p16^INK4a^ positive cells further accumulated from day 5 to day 7 at least in glomerula consistent with spreading of the senescent phenotype in response to SASP mediated inflammation [5]. Although we cannot clearly distinguish the inflammatory response provoked by reperfusion injury and alloimmunity from that induced by senescent cells, our findings indicate that Rapa efficiently breaks the vicious cycle of senescence and inflammation. Remarkably, this capability is apparently not only due to suppression of inflammation but can also be attributed to direct antisenescent properties.

A downside of all immunosuppressant agents is an inhibitory effect on the physiologically occurring clearance of senescent cells by natural killer cells, monocytes/macrophages, and T cells orchestrated by the SASP in immunocompetent individuals [4,42]. However, our data extends existing knowledge that immunosuppressants differ in their ability to induce senescence. While CNI related IF/TA has been linked to premature cellular senescence [3,13], early conversion to mTORi decreased IF/TA [43]. Interestingly, of the two CNI in clinical use, only Cyclosporin A, but not Tacrolimus, triggered senescence in cultured human renal tubular cells [14]. Thus, by combining several properties that can limit premature cellular senescence, mTORi such as Rapa occupy a unique position in the immunosuppressive armamentarium after organ transplantation.

Mechanistically, there are multiple links between mTOR and cellular senescence. mTOR is a serin/threonine protein kinase that is contained in two different multi-protein complexes called mTOR complex 1 (mTORC1) and 2 (mTORC2) with distinct upstream modulators and downstream effectors [22]. While mTORC1 controls cell growth by integrating energy availability, oxygen supply, and growth factor stimulation, mTORC2 is involved in the regulation of survival and proliferation in response to growth factors [22]. Rapa allosterically inhibits phosphorylation of several but not all mTORC1 targets resulting in, amongst others, reduced protein synthesis via p70S6 kinase 1 (p70S6K) and increased autophagy via Unc-51 like autophagy activating kinase 1 (ULK1) [22]. It has been suggested that reduced mRNA translation as a consequence of mTORC1 inhibition can limit oxidative stress [22], a major contributor to the development of premature cellular senescence [5] related to preservation/reperfusion injury in kidney transplantation [4,44]. Furthermore, induction of autophagy promoting clearance of damaged proteins and organelles in addition to restriction of protein synthesis can prevent proteotoxic stress and the unfolded protein response leading to reduced cellular senescence [17]. Rapa does not directly inhibit mTORC2 but rather stimulates mTORC2 activity through release of a negative feedback loop [22]. Rapa mediated blockade of mTORC1 with reciprocal activation of mTORC2 can not only activate autophagy and reduce cellular senescence but also recruit prosurvival pathways resulting in reduced apoptosis [20]. Although we did not assess apoptosis in our model, this mechanism might also have contributed to rapid recovery of tubular function. Besides its effect on cell fates, Rapa also favors an undifferentiated state of progenitor cells [20,45] and could promote ‘stemness’ and stem cell function [22,23] thereby preserving organ plasticity and regeneration potential beyond the peritransplant period conferring resistance to future injuries that could accelerate IF/TA and compromise long-term graft function.

Our data suggests that the use of mTORi in *de novo* transplants does not impair regeneration after preservation/reperfusion injury despite the central role of mTORC1 for synthesis of proteins, lipids, and nucleotides needed for cell growth and division [22] when trough levels are kept in the lower range comparable to those established in the ELITE-Symphony Study [37]. Low-dose mTORi in combination with reduced CNI exposure has evolved as an immunologically safe but less nephrotoxic alternative to standard exposure CNI based regimens [46,47]. As presented here, Rapa treated animals showed less tubular dysfunction and creatinine clearance did not differ between both groups within the first week after transplantation. It can be expected that early protection from senescence by Rapa as observed in our short-term model will pay off with better graft function in the long run since senescence is a determinant of IF/TA^3^ that in turn is a major cause of kidney allograft failure [2].

Additional treatment strategies targeted at reducing cellular senescence early in the time course of transplantation and long-term are desirable. As a basis, a healthy life-style with regular physical exercise and dietary interventions should be promoted since this has been shown to improve metabolic parameters such as insulin sensitivity and lipid profile and to reduce DNA damage as well as oxidative stress as potential mechanisms to delay age related senescence [4,48]. Recently, a novel strategy to reduce the senescent burden by therapeutic elimination of senescent cells is emerging. Senolytic drugs clear senescent cells by overcoming apoptosis resistance conferred by senescent cell antiapoptotic pathway (SCAP) networks [49]. So far, hypothesis-driven approaches and drug screenings have identified several compounds with senolytic activity such as Navitoclax, Querticin, Dasatinib, Curcumin, FOXO4-related peptide, and others [4,49]. Some of these drugs have been shown to improve renal outcomes in pre-clinical studies [4,49] with the greatest body of evidence being available for Querticin and Dasatinib but lack of specificity for senescent cells and serious adverse effects associated with these compounds have obviated comparable studies in humans, yet. Transplantation, however, offers the unique opportunity to selectively treat an organ at risk for injury-induced premature cellular senescence without systemic effects during the preservation period or immediately prior to implantation. This approach could also reset the already advanced senescent phenotype of kidneys from older donors that are more susceptible to preservation/reperfusion injury resulting in graft dysfunction and decreased graft survival [3,6,8,9,16,50]. Combination with interventions aimed at slowing down recurrent accumulation of senescent cells after transplantation such as life-style modifications and use of mTORi for rejection prophylaxis might be most effective to protect from IF/TA and optimize graft survival though cellular senescence is not the only mechanism of renal allograft damage [1].

Taken together, our findings imply that even low doses of the mTOR inhibitor Rapa started in the immediate posttransplant period effectively prevent the development of premature cellular senescence originating from transplantation related stress in all compartments of the renal allograft. mTOR inhibition was well tolerated with respect to regeneration, graft function, metabolism, and growth. Thus, the regenerative capacity of the transplanted kidney might be maintained best by mTORi in comparison to all other available immunosuppressants providing a rationale for the combination of mTORi with reduced CNI exposure to improve long-term graft function and survival.

## Supporting information

S1 Checklist (PDF)

S1 Fig Inflammatory infiltrate after kidney transplantation in vehicle and Rapa treated rats. (A) ED1 and (B) ED2 positive macrophages in the outer medulla calculated as percentage of positive interstitial cells by immunohistochemistry. (C) CD8 positive T-cells in the outer medulla calculated as percentage of positive interstitial cells by immunohistochemistry. n = 3. *P<0.05, **P<0.01, ***P<0.001.
(TIF)

S1 File (XLSX)
