# Supplementary figures and images for: The mTOR inhibitor Rapamycin protects from premature cellular senescence early after experimental kidney transplantation
Source: PLoS One. 2022 Apr 21;17(4):e0266319. doi: 10.1371/journal.pone.0266319 (PMC9022825; doi:10.1371/journal.pone.0266319)

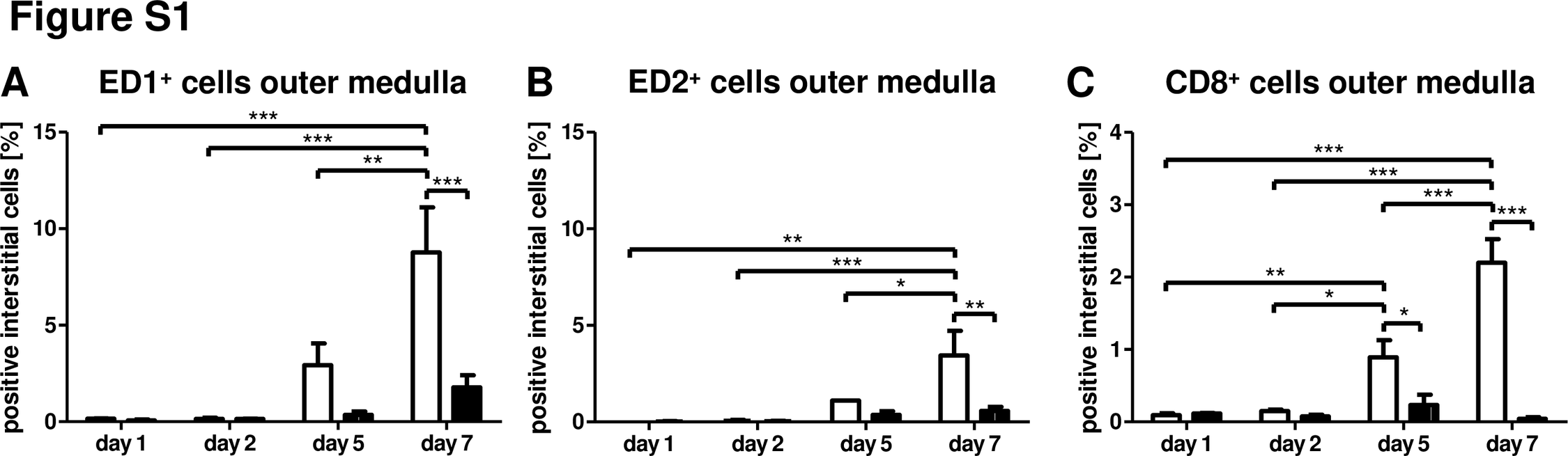

Supplement: S1 Fig — (A) ED1 and (B) ED2 positive macrophages in the outer medulla calculated as percentage of positive interstitial cells by immunohistochemistry. (C) CD8 positive T-cells in the outer medulla calculated as percentage of positive interstitial cells by immunohistochemistry. n = 3. *P<0.05, **P<0.01, ***P<0.001. (TIF) [file pone.0266319.s002.tif]
